# Co-Occurrence of Psoriasis and Asthma in the Pediatric Population: A Systematic Review and Meta-Analysis

**DOI:** 10.3390/jcm13226991

**Published:** 2024-11-20

**Authors:** Mateusz Mleczko, Agnieszka Gerkowicz, Dorota Krasowska

**Affiliations:** Department of Dermatology, Venerology and Pediatric Dermatology, Medical University of Lublin, 11 Staszica St., 20-081 Lublin, Poland

**Keywords:** asthma, psoriasis, children, meta-analysis

## Abstract

**Introduction**: The risk of asthma in patients with psoriasis and that of psoriasis in patients with asthma have increased, but relevant data for the pediatric population are lacking. Therefore, we performed a meta-analysis to assess the pooled association between psoriasis and asthma in children and adolescents. **Methods**: We conducted an extensive search of the medical literature databases through to July 2024. The estimated risk ratios (RRs) and corresponding 95% confidence intervals (CIs) were computed. **Results**: Three studies, involving 5310 children with psoriasis and 1,539,029 control participants, were included to evaluate the incidence of asthma in children with psoriasis. The meta-analysis indicated a significantly increased risk of asthma in children with psoriasis [RR 1.38 (95% CI, 1.28–1.49)]. Additionally, two studies involving 104,369 asthmatic children and 1,539,029 controls were included to evaluate the incidence of psoriasis in children with asthma. The meta-analysis indicated a significant increase in the risk of psoriasis in children with asthma [RR 1.17 (95% CI, 0.70–1.95)]. **Conclusions**: This meta-analysis offers evidence supporting the association between psoriasis and asthma in pediatric populations. Therefore, physicians should make patients aware of the connection between these two chronic diseases.

## 1. Introduction

Psoriasis is a common chronic inflammatory skin disease which can affect people of all ages [1]. The mean age of onset of pediatric psoriasis is between 8 and 11 years, and the prevalence increases with age, estimated at 0.12% at the age of 1 year to 1.2% at the age of 18 years [2]. Psoriasis is often mistaken for eczema, suggesting that the prevalence of pediatric psoriasis may be higher [3]. Similar to psoriasis, asthma is a chronic inflammatory disorder of the airways that affects all age groups [4]; however, the prevalence of asthma is higher in children aged 0–17 years (9.5%) than in adults aged 18 and over (7.7%) [5]. Moreover, a significant increase in the reported prevalence of asthma has been observed in the past few years [6].

Psoriasis is a chronic immune-mediated disease in which the inflammatory response is supported by T helper (Th)1 and Th17 lymphocytes; furthermore, Th22 activation also plays an important role, resulting in the activation of the interleukin (IL)-23/17 signaling pathway and tumor necrosis factor-α (TNF-α) [7,8,9]. The differentiation and activation of Th17 cells are caused by the presence of cytokines, including IL-1β, IL-6, transforming growth factor β, and IL-23 [10]. In contrast to psoriasis, asthma results from chronic inflammation of the airways, and it is believed to be caused primarily by Th2 lymphocytes and mediated by Th2 cytokines such as interleukin (IL)-5, IL-4, and IL-13 [11]. However, Th2 inflammation is responsible for only approximately 50% of mild-to-moderate asthma and a large portion of severe asthma [12].

**Figure 1:**
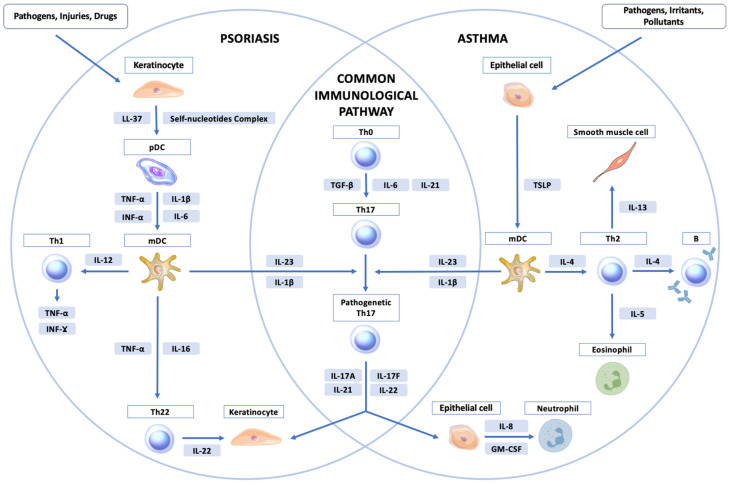
Potential common pathogenetic pathway of psoriasis and asthma. DC: dendritic cell; INF-α, -γ: interferon-α, -γ; IL-1β, -4, -5, -6, -8, -12, -13, -17A, -17F, -21, -22, -23: interleukin-1β, -4, -5, -6, -8, -12, -13, -17A, -17F, -21, -22, -23; LL-37: peptide derived from hCAP with 37 amino acids and two leucines at its N-terminal; mDC: myeloid dendritic cell; pDC: plasmocytoid dendritic cell; Th0, -1, -2, -17: T helper cell 0, -1, -2, -17; TNF-α: tumor necrosis factor-α; TGF-β: transforming growth factor-β; TSLP: thymic stromal lymphopoietin.

Recent studies have demonstrated the involvement of Th17 lymphocytes in the pathogenesis of asthma mediated by IL-17A and TNF-α [13,14,15]. Th17 produces IL-17A and IL-17F, which are elevated in some individuals with asthma and may be responsible for the recruitment of antigen-induced neutrophils into the airways and the enhancement of Th2 cell-mediated eosinophil recruitment into the airways, as seen in steroid-resistant asthma [16,17]. Moreover, while IL-17 production has been shown to be associated with the severity and exacerbation of asthma, the data remain unclear [18,19,20]. Although Th1- and Th2-driven diseases were previously thought to be inversely related, studies have shown an increased risk of asthma in relation to some Th1-driven diseases [21,22,23]. As demonstrated in this meta-analysis, an increased risk of asthma occurs in psoriasis (and vice versa). Figure 1 shows the potential common pathogenetic pathways of psoriasis and asthma.

Psoriasis and asthma in children, especially those with concomitant diseases, can contribute to the physical and psychosocial burdens of the diseases and can negatively impact quality of life [24,25]. In previous studies, it has been shown that children with psoriasis also show increased rates of cardiometabolic diseases [26], obesity [27], gastrointestinal diseases [28], and mental health disorders [29], when compared to those without pediatric psoriasis. In turn, children with asthma also present increased rates of other atopic diseases [30,31], obesity [32,33], gastrointestinal diseases [34], and mental health disorders [35]. Moreover, single epidemiological studies have shown an increased risk of asthma in pediatric psoriasis [36,37,38] and, likewise, psoriasis in asthmatic children [39,40].

When considering that the conclusions of single studies may be unreliable, we performed a meta-analysis focused on asthma in pediatric psoriasis and psoriasis in pediatric asthma in order to determine the potential association between these two diseases. The primary objective of this study is to assess the risk of psoriasis in pediatric patients with asthma and of asthma in those with psoriasis. This study updates and supplements a previous meta-analysis [41] on asthma in pediatric patients with psoriasis vs. controls by including a new meta-analysis on psoriasis in pediatric patients with asthma vs. controls.

## 2. Materials and Methods

### 2.1. Data Sources and Search Strategy

We conducted a thorough systematic review and meta-analysis following the Preferred Reporting Items for Systematic Reviews and Meta-Analyses (PRISMA) guidelines and pre-registered the protocol in the International Prospective Register of Systematic Reviews (PROSPERO) platform with the registration number CRD42024556894. To evaluate the risk of asthma in pediatric patients with psoriasis and the risk of psoriasis in children with asthma, we thoroughly searched the PubMed/MEDLINE, Embase, and Cochrane Library databases. The search utilized medical subject headings (MeSH) and incorporated the terms (asthma) AND (psoriasis) AND (children). The search of the medical literature databases was conducted through to 4 July 2024. There were no language, population, or publication date limits.

### 2.2. Selection Criteria and Eligibility

We imported all the citations from the databases into Zotero and removed any duplicate entries. Two independent reviewers screened the titles and abstracts to determine eligibility. Articles meeting the inclusion criteria underwent a full-text examination for potential inclusion in this review. The inclusion criteria for the meta-analysis were as follows: (1) cohort, case-control, or cross-sectional design; (2) analysis of the risk of asthma in children with psoriasis or the risk of psoriasis in children with asthma; and (3) inclusion of a reference group. The exclusion criteria were as follows: (1) cell or animal models; (2) insufficient data; and (3) reviews, comments, abstracts, and case reports. The studies were divided into the following two groups: (1) analysis of the risk of asthma in children with psoriasis; and (2) analysis of the risk of psoriasis in children with asthma.

### 2.3. Data Extraction

Two independent reviewers extracted the following information from eligible studies: first author; publication date; study design; diagnostic criteria for asthma and psoriasis; number of asthma cases in psoriasis and controls; number of psoriasis cases in asthma and controls; and age of patients. Conflicts were resolved through open and constructive dialogue.

### 2.4. Risk of Bias Assessment

The quality of cohort studies was assessed using the Newcastle–Ottawa Scale [42] by two independent reviewers. For all the studies, selection, comparability, and outcome were assessed. The number of scored points varied from 0 to 9 (<4, low quality; 4–5, medium quality; >6, high quality).

For cross-sectional studies, we used the recommended tools of the Agency for Healthcare Research and Quality (AHRQ), which consists of 11 items, where each item has “yes”, “no”, and “unclear” as responses (scored as 1, 0, and 0 points, respectively). The number of scored points varied from 0 to 11 (<4, low quality; 4–7, average quality; >7, high quality).

### 2.5. Statistical Analysis

The data from the study were analyzed using SPSS 29.0 (IBM SPSS Statistics for Windows, version 29.0. Armonk, NY, USA). To evaluate the risk of asthma in children with psoriasis and the risk of psoriasis in children with asthma, estimated risk ratios (RRs) and 95% confidence intervals (CIs) were calculated. Heterogeneity among the included studies was evaluated using the I^2^ statistic. If *p* > 0.1 and I^2^ ≤ 50%, the fixed-effect model was used; meanwhile, if I^2^ > 50%, the random-effect model was used. Additionally, when there was excessive heterogeneity, a sensitivity analysis was conducted, and recalculations were performed after excluding studies that displayed significant heterogeneity. To address potential publication bias, we employed a funnel plot and Egger’s regression to assess the presence of this bias. If the *p*-value is >0.05, it indicates no publication bias; conversely, if it is <0.05, adjustments will be made using the trimming method. Results were deemed statistically significant when the *p*-value was less than 0.05. 

## 3. Results

### 3.1. Characteristics of Identified Studies

**Figure 2:**
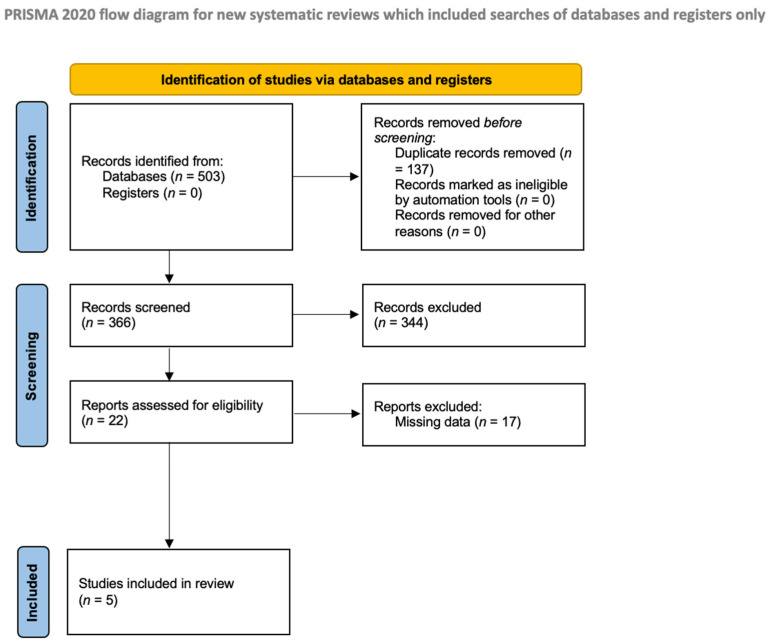
Flow diagram of the study inclusion process.

Figure 2 outlines the search methodology and literature review process. A total of 503 articles were identified for eligibility. The titles and abstracts of 22 articles met the eligibility criteria for a full-text review. After further screening, five studies met the inclusion criteria for the systemic review.

These five studies were conducted between 2015 and 2023, and a total of 2,999,631 subjects were included in the meta-analysis, the characteristics of which are presented in Table 1 for patients with psoriasis compared with controls and in Table 2 for patients with asthma compared with controls.

As for ethnicity, three studies investigated European populations, and one investigated an Asian population. According to Galili’s study, the country of origin was classified as the father’s or grandfather’s country of birth, with the following results: Africa and Asia (18.62%); America, Australia, and Europe (22.89%); Israel (9.38%); and Russia and former USSR (49.11%). Socioeconomic status was analyzed in two studies, based on the place of residence or income. The studies included patients of various ages, but all were <19 years old and one study provided detailed incidences of asthma at different ages. Other adjusting factors included age, body mass index, comorbidities, medications, and number of siblings. Moreover, one study provided the incidence of asthma in the mild and the moderate-to-severe psoriasis groups.

### 3.2. Asthma in Psoriasis Patients

**Figure 3:**
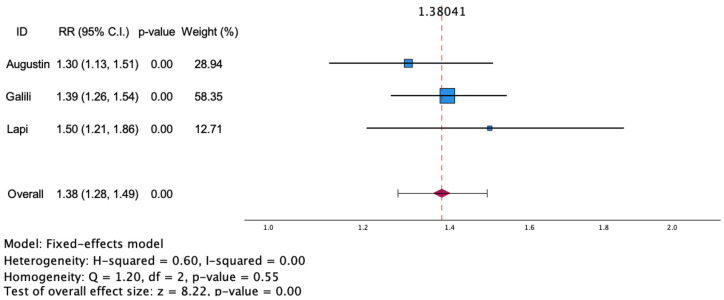
Estimated risk of asthma in patients with psoriasis. Square blue boxes represent the effect size of each study; horizontal lines represent 95% confidence intervals (CIs) of effect size; and diamond-shaped red figure represent the estimated overall effect size. Axis is shown using log scale [36,37,38].

The meta-analysis exploring the risk of asthma in patients with psoriasis included two population-based cross-sectional studies and one retrospective cohort study, with 5310 psoriasis patients and 1,180,825 controls. The estimated risk ratio of asthma in patients with psoriasis was 1.38 [95% CI (1.28, 1.49); I^2^ = 0%; *p* < 0.001]. As the I^2^ of the study was below 50%, which indicates low heterogeneity, it is reasonable to use the fixed-effect model. The RR of individual studies varied from 1.3 to 1.5. A forest plot of the risk of asthma in psoriasis patients is shown in Figure 3. The risk of asthma among patients with psoriasis could not be analyzed by the subgroup, due to the small number of included articles.

### 3.3. Psoriasis in Asthma Patients

**Figure 4:**
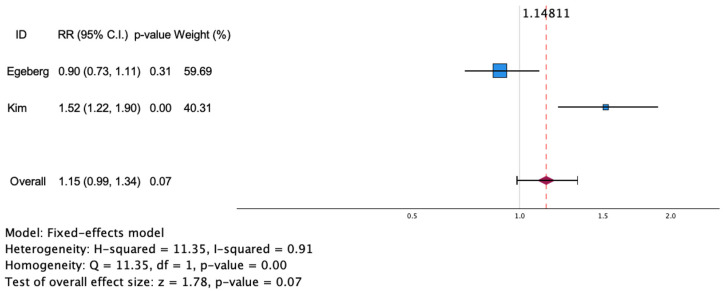
Estimated risk of asthma in patients with psoriasis. Square blue boxes represent the effect size of each study; horizontal lines represent 95% confidence intervals (CIs) of effect size; and diamond-shaped red figure represent the estimated overall effect size. Axis is shown using log scale [39,40].

The meta-analysis exploring the risk of psoriasis in patients with asthma included two retrospective cohort studies, with 104,369 asthma patients and 1,539,029 controls. The estimated risk ratio of psoriasis in patients with asthma was 1.17 [95% CI (0.70, 1.95); I^2^ = 91%; *p* = 0.56]. As the I^2^ of the study was below 50% (*p* = 0.07), which indicates homogeneity, it is reasonable to use the fixed-effect model. The RR of individual studies varied from 0.9 to 1.52. A forest plot of the risk of psoriasis in asthma patients is shown in Figure 4. The risk of psoriasis among patients with asthma could not be analyzed by subgroup, due to the small number of articles included.

### 3.4. Quality Assessment and Publication Bias

Table 3A shows the risk of bias based on individual studies using the Newcastle–Ottawa Scale. Four studies were of high quality (≥7), while one was of moderate quality (4–6). Table 3B shows the risk of bias based on one study using the Agency for Healthcare Research and Quality (AHRQ), which was of high quality (≥7). In summary, all studies considered in this meta-analysis were of sufficient quality.

**Figure 5:**
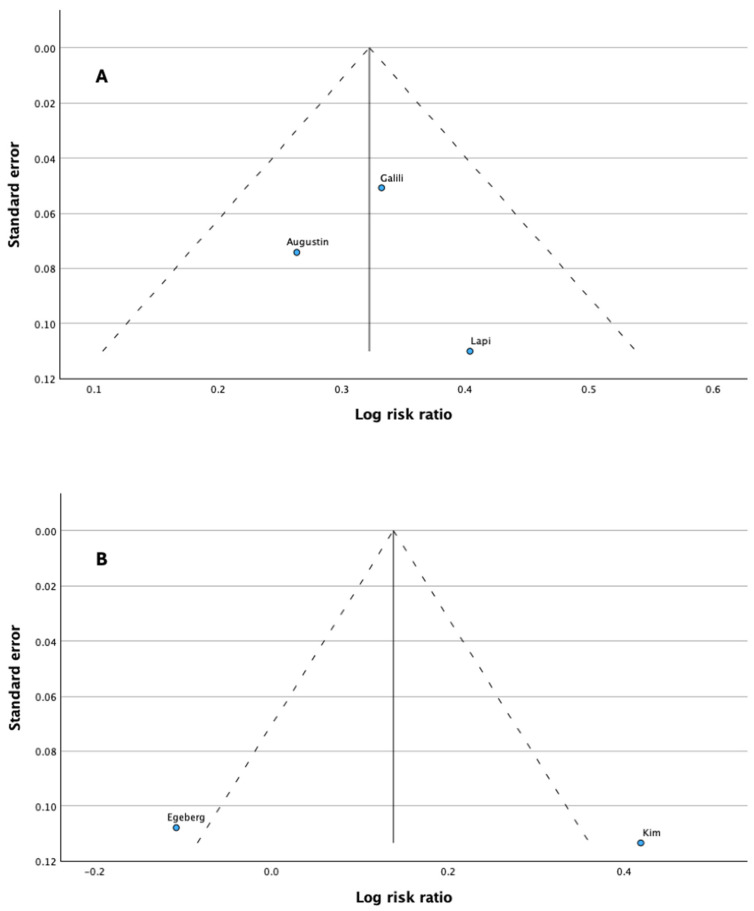
Funnel plot for publication bias in the selected studies. (**A**) Asthma in psoriasis patients compared with controls; (**B**) Psoriasis in asthma patients compared with controls. Blue dots represent primary studies; dashed lines represent 95% confidence intervals (CIs) of effect size; and solid lines represent estimated overall effect size [36,37,38,39,40].

Figure 5 provides a visual depiction of the funnel plots for each set of comparisons. In the studies, no significant asymmetry was observed, indicating that there was no publication bias. We assessed publication bias through the visual examination of a funnel chart and Egger’s regression test. Our analysis concluded that there was no significant publication bias in the literature regarding the two-way association between psoriasis and asthma (see Figure 4 and Figure 5). The visual results from the funnel chart, along with the findings from two Egger’s regression tests (*p* = 0.279), provide evidence that publication bias was not present. Note that Egger’s regression-based test cannot be computed when the number of studies is less than or equal to two.

## 4. Discussion

This systematic review and meta-analysis consisted of five studies, including two cross-sectional and three cohort studies, with a total of 2,999,631 subjects, providing clear evidence that children with psoriasis have a 1.38 risk ratio for asthma and children with asthma have a 1.17 risk ratio for psoriasis.

Similar relationships can be reached using data concerning adults [43]. However, substantial differences might be seen between adult- and childhood-onset asthma. Childhood asthma significantly differs from adult asthma, due to variations in the immunological phenotype [44]. These phenotypes may feature distinct immunological signatures, including a Th2/Th17 reaction in asthma [45]. This is probably not the only pathogenetic pathway common to psoriasis and asthma, as supported by the initial clinical trials targeting IL-23 and IL-17 in asthma which yielded unfavorable results [46,47]. Challenges have been posed to the traditional Th1/Th2 paradigm in recent years, which asserts that diseases driven by Th1 and Th2 cells are inversely related due to immune cross-regulation [48]. Research has shown that Th1- and Th2-driven diseases can coexist, with a parallel increase in the prevalence of asthma and certain Th1-driven diseases, including psoriasis [23,49]. Recent evidence suggests that inflammation-independent processes also contribute to the pathogenesis of asthma [12]. Further research is needed to explore potentially unifying inflammatory pathways in the context of psoriasis and asthma.

In relation to the above, the resemblance in the immunophenotype between the Th-17-high asthma phenotype and psoriasis is supported by genetic studies in which, for example, genes expressed in asthma patients with high IL-17 levels were common with those reported to be altered in psoriatic lesions, including genes regulating epithelial barrier function and defensive mechanisms [50]. The genetic contribution to the association study in children revealed single-nucleotide polymorphisms based on genome-wide association studies (GWASs) which are common in the context of both asthma and psoriasis susceptibility [51,52,53]. However, Lonnberg et al. have reported that there was no difference between twins with psoriasis and those without psoriasis but with susceptibility to asthma due to familial risk factors, indicating that the risk of familial disease is more important in the development of asthma; in particular, this may hide or attenuate the genetic risk factors [54].

One of the main problems with all studies assessing the link between psoriasis and asthma is the lack of consistent data on the status of some common risk factors. It has been confirmed that the risks of asthma in psoriasis patients and psoriasis in asthma patients may be associated with common risk factors [55]. Environmental factors, such as smoking status, metabolic syndrome, and medications, are more likely to play a role in adult asthma pathogenesis [56]. Adult-onset asthma is often linked to occupation [57], frequently occurs in non-atopic individuals, and is associated with a poorer prognosis [58]. Additionally, it may be mistaken for chronic obstructive pulmonary disease (COPD) or overlap with its symptoms [59]. The lack of adjustment for common risk factors may have influenced the results of this meta-analysis. In two studies, the risk of asthma in children with psoriasis was assessed while taking age, sex, country of origin, socio-economic status, number of siblings, comorbidities and body mass index into account; however, the increased risk remained consistent after an adjustment for possible confounding factors [37,40]. Moreover, the association between adolescent psoriasis and asthma was observed for only moderate-to-severe psoriasis [37]. This may be due to the limited and likely underestimated incidence and prevalence data regarding juvenile psoriasis, as well as because patients with mild disease frequently do not seek medical evaluation or may be misdiagnosed [60].

In contrast in Augustin’s study, children with psoriasis show increased comorbidity associated with obesity. In particular, the incidence rate of the components of the metabolic syndrome, including obesity (7.08%), hyperlipidemia (1.14%), hypertension (0.91%), and diabetes (0.61%), is significantly higher than in children without psoriasis. In contrast, children with atopic eczema often experience increased rates of atopic comorbidities, such as allergic rhinitis and bronchial asthma. The prevalence of juvenile psoriasis increases constantly from early childhood up to the age of 18, whereas the prevalence of atopic dermatitis decreases over the years, underlining the essential differences between these two conditions [36]. However, in light of the above observations, it has been evidenced that pediatric patients with psoriasis are at risk of developing metabolic disorders and related comorbidities, including bronchial asthma. Moreover, weight loss has been shown to substantially improve the clinical status of patients with asthma [61]. Other important environmental risk factors for these two diseases in children include ethnicity, low socioeconomic status, air pollution, allergies, infections, and dysbiosis [55]. The role of drugs in the pathogenesis of both diseases in children is still unknown. The Egeberg study found that pediatric asthma significantly increased the risk of overall and mild psoriasis but not severe psoriasis [39]. It remains unclear whether this observation is due to the low number of events or the impact of asthma treatment on psoriasis severity, necessitating further investigation. Other common risk factors may also be at play.

There are a few limitations in this meta-analysis. First, the meta-analysis did not contain detailed information on confounding variables, including ethnicity, body mass index (BMI), dietary preference, drug history, family history of systemic diseases, and socioeconomic status, all of which can be risk factors for psoriasis and asthma. Second, in relation to the above, there was a lack of information on the reported asthma phenotype. Third, only published data were retrieved, there may still be publication bias in this meta-analysis, although no significant asymmetry was identified in the funnel plots. The prevalence rates are only reflective of patients who seek medical care, and children with psoriasis and atopic eczema might not seek treatment. Fourth, both asthma and psoriasis are complex diseases that may be misdiagnosed, and the diagnostic criteria for asthma or psoriasis were not entirely consistent between the selected studies. Moreover, comorbidities might have been under-reported. Considering these limitations, the results of this meta-analysis should be interpreted with caution.

## 5. Conclusions

This review delivered clear evidence for the association between psoriasis and asthma. The ongoing increase in psoriasis and asthma rates among children confirms these conditions as significant public health issues. The risk of psoriasis should be considered in asthma patients (and vice versa) in order to effectively implement preventive and therapeutic strategies that reduce the lifetime risk of disease progression. While the introduction of biological agents presents new opportunities for psoriasis and asthma treatment, early detection and intervention continue to be the most important methods of medical care.

Future research should focus on elucidating the underlying pathophysiological mechanisms that link psoriasis and asthma in children, as this may help in the development of appropriate preventative approaches; facilitate the selection of comorbidity-guided personalized treatments; and contribute to development of novel therapies for both psoriasis and asthma, including intrinsically therapeutic nanoparticles [62]. Investigating the genetic, immunological, and environmental factors could provide deeper insights into the shared etiology of these conditions. Understanding the roles of lifestyle modifications, comorbidities, and treatment responses will be crucial for developing comprehensive care strategies tailored to pediatric patients suffering from these chronic inflammatory diseases. Moreover, a future study with homogeneous or classified endotypes of asthma and psoriasis could help to identify the potential predictive factors for these diseases.

## Figures and Tables

**Table 1:** Characteristics of the three studies on the prevalence of asthma in psoriasis patients compared with controls. ICD-10: The International Classification of Diseases, 10th revision; ICD-9-CM: The International Classification of Diseases, 9th Revision, Clinical Modification; NA: not available.

Author	Year	Country	Study Design	Diagnosis Criteria Asthma/Psoriasis	Cases, No. with Asthma/Total No.	Controls, No. with Asthma/Total No.	Included Age	Adjustment
Augustin [36]	2015	Germany	Population-based cross-sectional	ICD-10 (detailed code has not been provided)/ICD-10 code L40	160/1313	27,319/291,868	0–18	NA
Galili [37]	2020	Israel	Population-based cross-sectional	Clinically by relevant specialist (pulmonologist/dermatologist)	345/3112	70,636/884,653	16–18	age, sex, country of origin, number of siblings, body mass index, and socioeconomic status
Lapi [38]	2023	Italy	Retrospective cohort	ICD-9-CM (detailed code has not been provided)/ICD-9-CM code 696	97/885	315/4304	0–18	NA

**Table 2:** Characteristics of the two studies on the prevalence of psoriasis in asthma patients compared with controls. ICD-10: The International Classification of Diseases, 10th Revision. * The meta-analysis included ages 0–18.

Author	Year	Country	Study Design	Diagnosis Criteria Psoriasis/Asthma	Cases, No. with Psoriasis/Total No.	Controls, No. with Psoriasis/Total No.	Included Age	Adjustment
Egeberg [39]	2015	Denmark	Retrospective cohort	ICD-10 codes L40, M070-M073/ICD-10 code J45	87/21,725	6499/1,456,385	6–14	age, sex, comorbidities, and medication
Kim [40]	2019	Korea	Retrospective cohort	ICD-10 code B02/ICD-10 codes J45–46	771/167,693	589/167,693	All ages *	Age, sex, income, region of residence, hypertension, diabetes, and dyslipidemia

**Table 3:** Risk of bias and quality assessment of studies according to the Newcastle–Ottawa Scale (A) and Agency for Healthcare Research and Quality (B).

A	Study, Year	Selection	Comparability	Outcome	Rating
	Augustin, 2015 [36]	3	0	1	4
	Egeberg, 2015 [39]	4	2	3	9
	Kim, 2019 [40]	4	2	3	9
	Lapi, 2023 [38]	4	2	3	9
**B**	**Study, Year**	**Yes**	**No**	**Unclear**	**Rating**
	Galili, 2020 [37]	9	1	1	9

## Data Availability

All data are provided in the systematic review.
